# Supplementary material for: Ultra-Rare Variants Identify Biological Pathways and Candidate Genes in the Pathobiology of Non-Syndromic Cleft Palate Only
Source: Biomolecules. 2023 Jan 26;13(2):236. doi: 10.3390/biom13020236 (PMC9953608; doi:10.3390/biom13020236)

Figure S1. Principal component analysis (PCA) depicting the comparison between nsCPO and controls. The PCA plot showing the first two components based on common HapMap SNPs depicts two separated clusters, one including all Italians samples and one for Iranians samples as well due different geographic ancestry. Instead, no ancestral differences are detected in Italian nsCPO and controls.

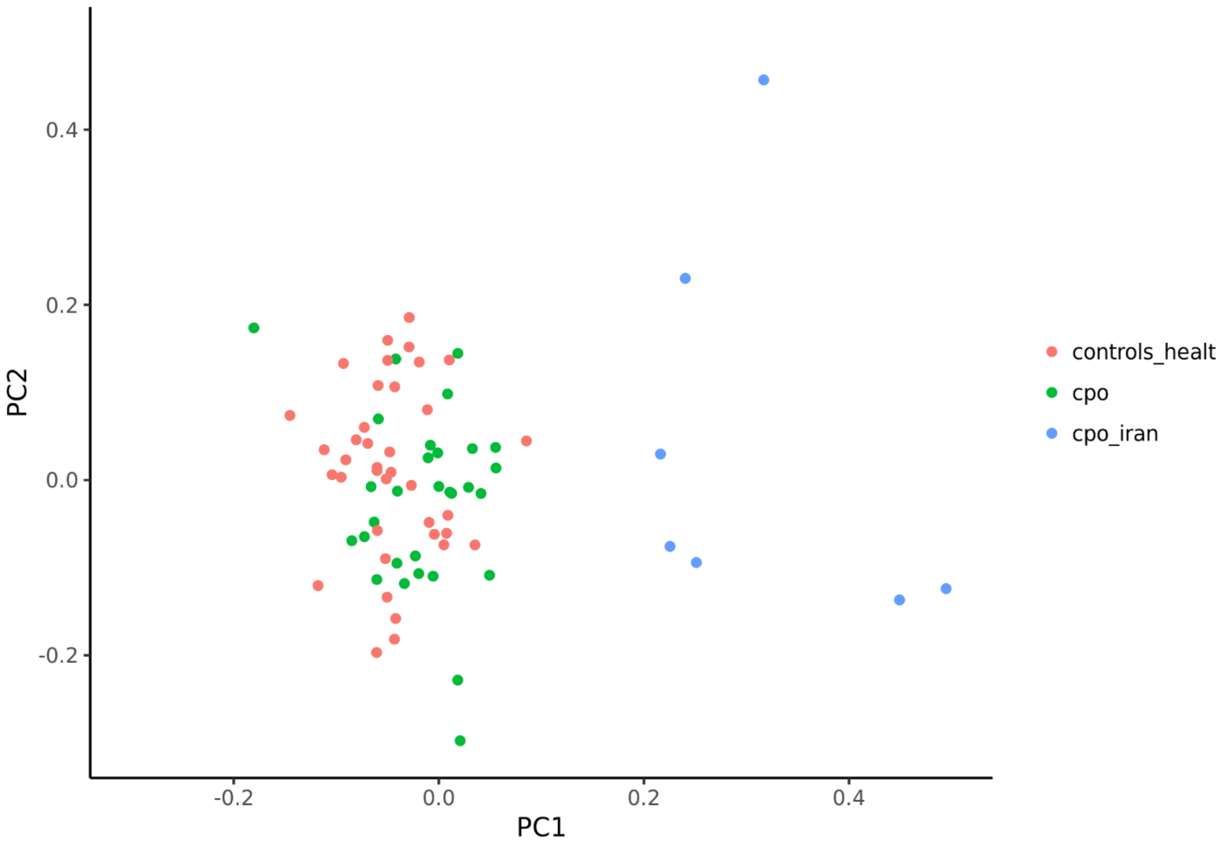

Supplement: Supplementary file 1 [file biomolecules-13-00236-s001.zip › Figure S1.pdf]
