# Supplementary material for: Ultra-Rare Variants Identify Biological Pathways and Candidate Genes in the Pathobiology of Non-Syndromic Cleft Palate Only
Source: Biomolecules. 2023 Jan 26;13(2):236. doi: 10.3390/biom13020236 (PMC9953608; doi:10.3390/biom13020236)

Figure S2. Number and size of ROHs detected in Italian and Iranian samples. Panel **A***)* shows the number of ROHs (NROH) identified in Italian cases and controls and in Iranian inbreed cases. The mean value of NROHs in Italian cases was 26.7; a similar number was found in Italian controls, 27.2. In Iranian cases, the NROH was higher according to an inbreeding model and was 38.6. The total size of ROHs larger than 1.5 Mb is shown in panel **B***)*. The SROHs follow the same shape as the NROHs (mean cases: 74.69 Mb, mean controls: 69.46 Mb, mean Iranian cases: 189.5 Mb).

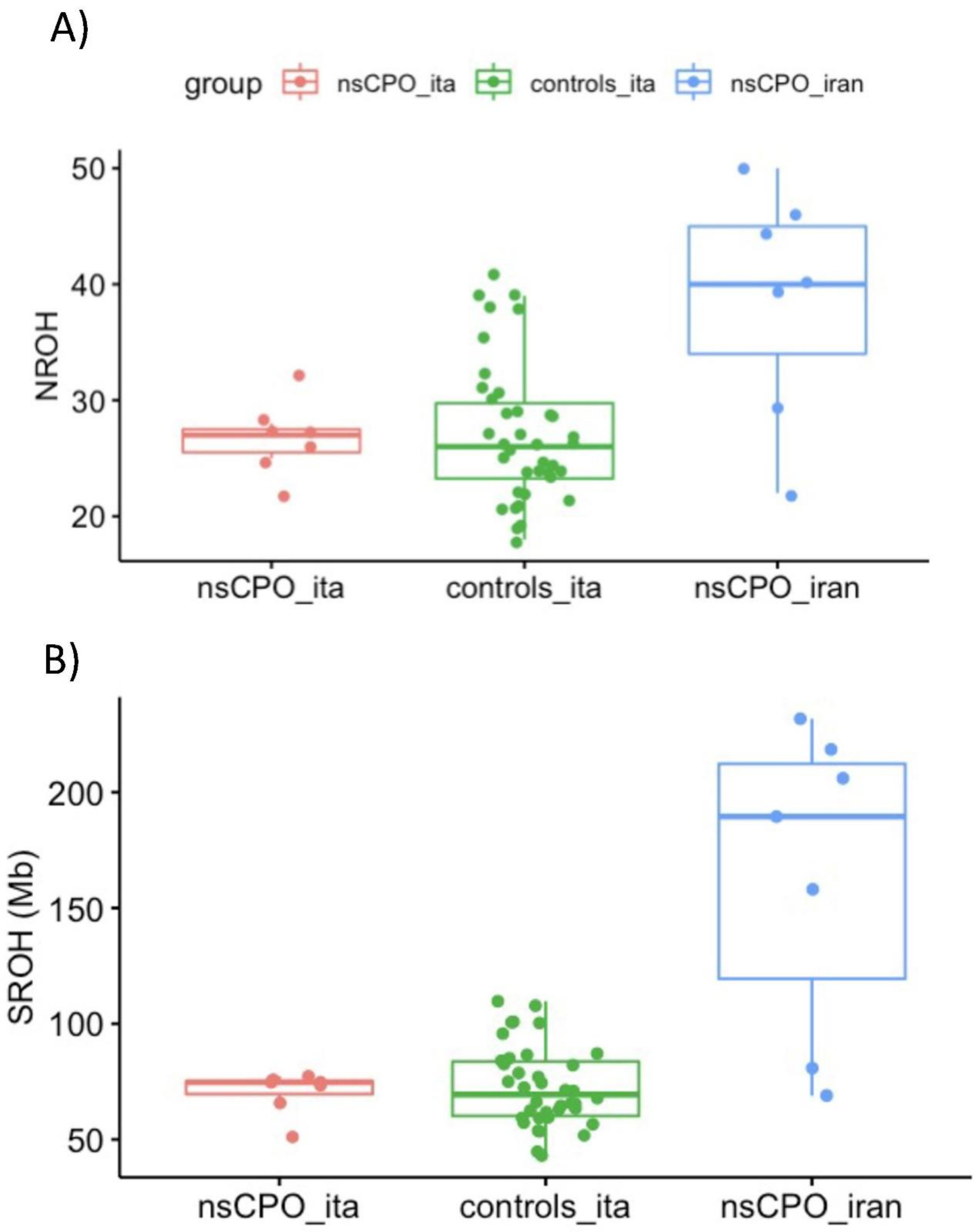

Supplement: Supplementary file 1 [file biomolecules-13-00236-s001.zip › Figure S2.pdf]
