# Supplementary material for: Ultra-Rare Variants Identify Biological Pathways and Candidate Genes in the Pathobiology of Non-Syndromic Cleft Palate Only
Source: Biomolecules. 2023 Jan 26;13(2):236. doi: 10.3390/biom13020236 (PMC9953608; doi:10.3390/biom13020236)

Figure S4. Sanger sequencing of the rare damaging variants of GLI3 gene identified by WES. The panel reports chromatograms of probands (right side) and their parents.

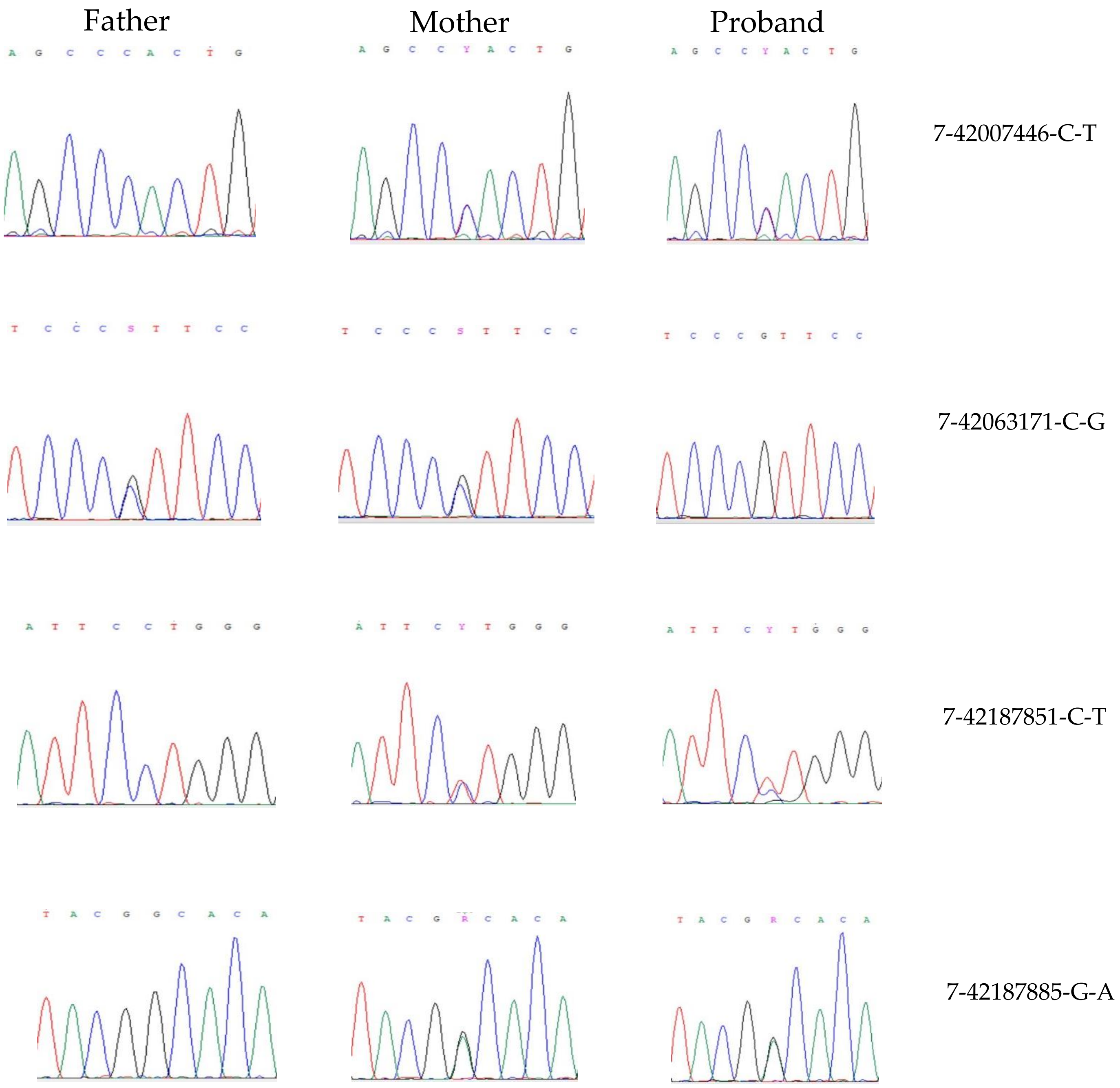

Supplement: Supplementary file 1 [file biomolecules-13-00236-s001.zip › Figure S4.pdf]
